# Supplementary material for: Non-linear machine learning models incorporating SNPs and PRS improve polygenic prediction in diverse human populations
Source: Commun Biol. 2022 Aug 22;5:856. doi: 10.1038/s42003-022-03812-z (PMC9395509; doi:10.1038/s42003-022-03812-z)
Supplement: Supplementary file 2 — Supplementary Information [file 42003_2022_3812_MOESM2_ESM.pdf]

Non-linear machine learning models incorporating SNPs and PRS improve polygenic prediction in diverse human populations:
Supplementary Materials

Elgart and Lyons et al.

Supplementary Note 1: Description of studies participating in the analysis and their Ethics Statements 2

HCHS/SOL 2

FHS 4

ARIC 5

CHS 6

MESA 7

CARDIA 8

JHS 9

CFS 11

Supplementary Figures 13

Supplementary Figure 1. ***Performance of linear PRSice, LDpred2, and lassosum models.*** 13

Supplementary Figure 2. ***Heritability of Phenotypes by Race/Ethnicity.*** 14

Supplementary Tables 15

Supplementary Table 1. ***Summary statistics of phenotypes in the testing dataset.*** 15

Supplementary Table 2. ***Summary statistics of phenotypes used in a secondary analysis in which the training dataset excluded related individuals*** 17

Supplementary Table 3. ***Excluding relatives from the training and validation datasets does not significantly change results*** 18

Supplementary Table 4. ***Selected parameters in cross-validation in the main multi-ethnic analysis for PRS and XGBoost***. 19

Supplementary Table 5***. Secondary analysis comparing the ensemble model with a standard linear PRS model using the same potential SNP set.*** 19

Supplementary Table 6. ***Comparison of LASSO SNP selection to random SNP selection for the XGBoost Alone and XGBoost with PRS models.*** 20

Supplementary Table 7. ***Secondary Analysis of Lassosum SNP selection for LDL Cholesterol.*** 20

Supplementary Table 8. ***Clumping the SNPs prior to performing the XGBoost ensemble model does not significantly change results.*** 20

Supplementary Table 9. ***Results of the PRS model without including the genetic PCs as covariates.*** 21

Supplementary Table 10. ***Results of the LASSO model when performing cross-validation for the optimal regularization term with respect to the LASSO loss function, rather than the joint-training scheme with XGBoost.*** 21

Supplementary Table 11. ***Number of SNPs selected through cross-validation in the race/ethnic-specific XGBoost models.*** 21

Supplementary Table 12: ***Phenotypes used in the analyses and their codes*** 22

Supplementary Table 13. ***Number of variants overlapping between the TOPMed genotype data after QC and filtering and the GWAS data.*** 24

Supplementary References 25

Supplementary Note 1: Description of studies participating in the analysis and their Ethics Statements

HCHS/SOL

The Hispanic Community Health Study/Study of Latinos (dbGaP accession phs000810) is a community-based longitudinal cohort study of 16,415 self-identified Hispanic/Latino persons aged 18–74 years and selected from households in predefined census-block groups across four US field centers (in Chicago, Miami, the Bronx, and San Diego). The census-block groups were chosen to provide diversity among cohort participants with regard to socioeconomic status and national origin or background [1,2]. The HCHS/SOL cohort includes participants who self-identified as having a Hispanic/Latino background; the largest groups are Central American (n = 1,730), Cuban (n = 2,348), Dominican (n = 1,460), Mexican (n = 6,471), Puerto Rican (n = 2,728), and South American (n = 1,068). The HCHS/SOL baseline clinical examination occurred between 2008 and 2011 and included comprehensive biological, behavioral, and sociodemographic assessments. Visit 2 took place between 2014 and 2017, which re-examined 11,623 participants

from the baseline sample. Visit 3 has started in 2020 and will last 3 years. In addition to clinic visit, participants are contacted annually to assess clinical outcomes. The study was approved by the Institutional Review Boards at each participating institution and written informed consent was obtained from all participants.

Ethics statement: This study was approved by the institutional review boards (IRBs) at each field center, where all participants gave written informed consent, and by the Non-Biomedical IRB at the University of North Carolina at Chapel Hill, to the HCHS/SOL Data Coordinating Center. All IRBs approving the study are: Non-Biomedical IRB at the University of North Carolina at Chapel Hill. Chapel Hill, NC; Einstein IRB at the Albert Einstein College of Medicine of Yeshiva University. Bronx, NY; IRB at Office for the Protection of Research Subjects (OPRS), University of Illinois at Chicago. Chicago, IL; Human Subject Research Office, University of Miami. Miami, FL; Institutional Review Board of San Diego State University. San Diego, CA.

Acknowledgements: The Hispanic Community Health Study/Study of Latinos is a collaborative study supported by contracts from the National Heart, Lung, and Blood Institute (NHLBI) to the University of North Carolina (HHSN268201300001I / N01-HC-65233), University of Miami (HHSN268201300004I / N01-HC- 65234), Albert Einstein College of Medicine (HHSN268201300002I / N01-HC-65235), University of Illinois at Chicago – HHSN268201300003I / N01- HC-65236 Northwestern Univ), and San Diego State University (HHSN268201300005I / N01-HC-65237). The following Institutes/Centers/Offices have contributed to the HCHS/SOL through a transfer of funds to the NHLBI: National Institute on Minority Health and Health

Disparities, National Institute on Deafness and Other Communication Disorders, National Institute of Dental and Craniofacial Research, National Institute of Diabetes and Digestive and Kidney Diseases, National Institute of Neurological Disorders and Stroke, NIH Institution-Office of Dietary Supplements.

FHS

The Framingham Heart Study (dbGaP accession phs000007) began in 1948 with the recruitment of an original cohort of 5,209 men and women (mean age 44 years; 55 percent women). In 1971 a second generation of study participants was enrolled; this cohort (mean age 37 years; 52% women) consisted of 5,124 children and spouses of children of the original cohort. A third-generation cohort of 4,095 children of offspring cohort participants (mean age 40 years; 53 percent women) was enrolled in 2002-2005 and are seen every 4 to 8 years. Details of study designs for the three cohorts are summarized elsewhere [3–5]. At each clinic visit, a medical history was obtained, and participants underwent a physical examination. Only study participants consented for genetic and non-genetic data are included. FHS has been approved by the Boston University IRB.

Ethics statement: The Framingham Heart Study was approved by the Institutional Review Board of the Boston University Medical Center. All study participants provided written informed consent.

Acknowledgments: The Framingham Heart Study (FHS) acknowledges the support of contracts NO1-HC-25195, HHSN268201500001I and 75N92019D00031 from the National Heart, Lung and Blood Institute and grant supplement R01 HL092577-06S1 for this research. We also

acknowledge the dedication of the FHS study participants without whom this research would not be possible. Dr. Vasan is supported in part by the Evans Medical Foundation and the Jay and Louis Coffman Endowment from the Department of Medicine, Boston University School of Medicine.

ARIC

The Atherosclerosis Risk in Communities (ARIC) study (dbGaP accession phs000090) is a population-based prospective cohort study of cardiovascular disease sponsored by the NHLBI. ARIC included 15,792 individuals, predominantly European American and African American, aged 45-64 years at baseline (1987-89), chosen by probability sampling from four US communities. Cohort members completed three additional triennial follow-up examinations, a fifth exam in 2011-2013, a sixth exam in 2016-2017, and a seventh exam in 2018-2019. The ARIC study has been described in detail previously [6].

Ethics statement: The ARIC study has been approved by Institutional Review Boards (IRB) at all participating institutions: University of North Carolina at Chapel Hill IRB, Johns Hopkins University IRB, University of Minnesota IRB, and University of Mississippi Medical Center IRB. Study participants provided written informed consent at all study visits.

Acknowledgements: The Atherosclerosis Risk in Communities study has been funded in whole or in part with Federal funds from the National Heart, Lung, and Blood Institute, National Institutes of Health, Department of Health and Human Services (contract numbers HHSN268201700001I, HHSN268201700002I, HHSN268201700003I, HHSN268201700004I and

HHSN268201700005I). The authors thank the staff and participants of the ARIC study for their important contributions.

CHS

The Cardiovascular Health Study (dbGaP accession phs000287) is a population-based cohort study initiated by the NHLBI in 1987 to determine the risk factors for development and progression of cardiovascular disease (CVD) in older adults, with an emphasis on subclinical measures. The study recruited 5,888 adults aged 65 or older at entry in four U.S. communities and conducted extensive annual clinical exams between 1989-1999 along with semi-annual phone calls, events adjudication, and subsequent data analyses and publications. Additional data are collected by studies ancillary to CHS. In June 1990, four Field Centers (Sacramento, CA; Hagerstown, MD; Winston-Salem, NC; Pittsburgh, PA) completed the recruitment of 5201 participants. Between November 1992 and June 1993, an additional 687 African Americans were recruited using similar methods. Blood samples were drawn from all participants at their baseline examination and during follow-up clinic visits and DNA was subsequently extracted from available samples

Ethics statement: All CHS participants provided informed consent, and the study was approved by the Institutional Review Board of the University Washington.

Acknowledgements: The Cardiovascular Health Study was supported by contracts HHSN268201200036C, HHSN268200800007C, HHSN268201800001C, N01HC55222, N01HC85079, N01HC85080, N01HC85081, N01HC85082, N01HC85083, N01HC85086,

75N92021D00006, and grants U01HL080295 and U01HL130114 from the National Heart, Lung, and Blood Institute (NHLBI), with additional contribution from the National Institute of Neurological Disorders and Stroke (NINDS). Additional support was provided by R01AG023629 from the National Institute on Aging (NIA). A full list of principal CHS investigators and institutions can be found at CHS-NHLBI.org. The content is solely the responsibility of the authors and does not necessarily represent the official views of the National Institutes of Health.

MESA

The Multi-Ethnic Study of Atherosclerosis (dbGaP accession phs000209) is a study of the characteristics of subclinical cardiovascular disease (disease detected non-invasively before it has produced clinical signs and symptoms) and the risk factors that predict progression to clinically overt cardiovascular disease or progression of the subclinical disease [7]. MESA consisted of a diverse, population-based sample of an initial 6,814 asymptomatic men and women aged 45-84. 38 percent of the recruited participants were white, 28 percent African American, 22 percent Hispanic, and 12 percent Asian, predominantly of Chinese descent. Participants were recruited from six field centers across the United States: Wake Forest University, Columbia University, Johns Hopkins University, University of Minnesota, Northwestern University and University of California - Los Angeles. Participants are being followed for identification and characterization of cardiovascular disease events, including acute myocardial infarction and other forms of coronary heart disease (CHD), stroke, and congestive heart failure; for cardiovascular disease interventions; and for mortality. The first examination took place over two years, from July 2000 - July 2002. It was followed by five

examination periods that were 17-20 months in length. Participants have been contacted every 9 to 12 months throughout the study to assess clinical morbidity and mortality.

Ethics statement: All MESA participants provided written informed consent, and the study was approved by the Institutional Review Boards at The Lundquist Institute (formerly Los Angeles BioMedical Research Institute) at Harbor-UCLA Medical Center, University of Washington, Wake Forest School of Medicine, Northwestern University, University of Minnesota, Columbia University, and Johns Hopkins University.

Acknowledgments: The MESA projects are conducted and supported by the National Heart, Lung, and Blood Institute (NHLBI) in collaboration with MESA investigators. Support for MESA is provided by contracts 75N92020D00001, HHSN268201500003I, N01-HC-95159, 75N92020D00005, N01-HC-95160, 75N92020D00002, N01-HC-95161, 75N92020D00003, N01-HC-95162, 75N92020D00006, N01-HC-95163, 75N92020D00004, N01-HC-95164, 75N92020D00007, N01-HC-95165, N01-HC-95166, N01-HC-95167, N01-HC-95168, N01-HC-95169, UL1-TR-000040, UL1-TR-001079, and UL1-TR-001420. Also supported in part by the National Center for Advancing Translational Sciences, CTSI grant UL1TR001881, and the National Institute of Diabetes and Digestive and Kidney Disease Diabetes Research Center (DRC) grant DK063491 to the Southern California Diabetes Endocrinology Research Center.

CARDIA

The Coronary Artery Risk Development in Young Adults study (dbGaP accession phs000285) is a prospective multicenter study with 5,115 adults Caucasian and African American participants of

the age group 18-30 years at baseline, recruited from four centers at the baseline examination in 1985-1986 [8]. The recruitment was done from the total community in Birmingham, AL, from selected census tracts in Chicago, IL and Minneapolis, MN; and from the Kaiser Permanente health plan membership in Oakland, CA. Nine examinations have been completed in the years 0, 2, 5, 7, 10, 15, 20, 25 and 30, with high retention rates (91%, 86%, 81%, 79%, 74%, 72%, 72%, and 71%, respectively) and written informed consent was obtained in each visit.

Ethics statement: All CARDIA participants provided informed consent, and the study was approved by the Institutional Review Boards of the University of Alabama at Birmingham and the University of Texas Health Science Center at Houston.

Acknowledgements: The Coronary Artery Risk Development in Young Adults Study (CARDIA) is conducted and supported by the National Heart, Lung, and Blood Institute (NHLBI) in collaboration with the University of Alabama at Birmingham (HHSN268201800005I & HHSN268201800007I), Northwestern University (HHSN268201800003I), University of Minnesota (HHSN268201800006I), and Kaiser Foundation Research Institute (HHSN268201800004I). CARDIA was also partially supported by the Intramural Research Program of the National Institute on Aging (NIA) and an intra-agency agreement between NIA and NHLBI (AG0005).

JHS

The Jackson Heart Study (dbGaP accession phs000286) is a longitudinal investigation of genetic and environmental risk factors associated with the disproportionate burden of cardiovascular

disease in African Americans [9,10]. JHS is funded by the NHLBI and the National Institute on Minority Health and Health Disparities (NIMHD) and is an expansion of the ARIC study in its Jackson Field Center. At baseline, the JHS recruited 5306 African American residents of the Jackson Mississippi Metropolitan Statistical Area aged, approximately 6.6% of all African American adults aged 35-84 residing in the area. Participants were recruited via random sampling (17% of participants), volunteers (30%), prior participants in the Atherosclerosis Risk in Communities (ARIC) study (31%), and secondary family members (22%). Among these participants, approximately 3400 gave consent that allows genetic research. JHS has conducted three back-to-back clinical examinations (Exam 1, 2000-2004; Exam 2, 2005-2008; and Exam 3, 2009-2013), and a fourth clinical examination is underway. Participants are also contacted annually by telephone to update personal and health information including vital status, interim medical events, hospitalizations, functional status, and sociocultural information.

Ethics statement: The JHS study was approved by Jackson State University, Tougaloo College, and the University of Mississippi Medical Center IRBs, and all participants provided written informed consent.

Acknowledgements and Disclaimer: The Jackson Heart Study (JHS) is supported and conducted in collaboration with Jackson State University (HHSN268201800013I), Tougaloo College (HHSN268201800014I), the Mississippi State Department of Health (HHSN268201800015I) and the University of Mississippi Medical Center (HHSN268201800010I, HHSN268201800011I and HHSN268201800012I) contracts from the National Heart, Lung, and Blood Institute (NHLBI) and

the National Institute on Minority Health and Health Disparities (NIMHD). The authors also wish to thank the staffs and participants of the JHS.

The views expressed in this manuscript are those of the authors and do not necessarily represent the views of the National Heart, Lung, and Blood Institute; the National Institutes of Health; or the U.S. Department of Health and Human Services.

CFS

The Cleveland Family Study (CFS) was designed to examine the genetic basis of sleep apnea in 2,534 African-American and European-American individuals from 356 families. Index probands with confirmed sleep apnea were recruited from sleep centers in northern Ohio, supplemented with additional family members and neighborhood control families [{Redline1995}]. Four visits occurred between 1990 and 2006; in the first 3, data were collected in participants' homes while the last occurred in a clinical research center (2000 - 2006). Measurements included sleep apnea monitoring, blood pressure, anthropometry, spirometry, and other related phenotypes. Blood samples (overnight fasting, before bed and following an oral glucose tolerance test), nasal and oral ultrasound, and ECG were also obtained during the 4th exam. Institutional Review Board approval and signed informed consent was obtained for all participants.

Ethics statement: Cleveland Family Study was approved by the Institutional Review Board (IRB) of Case Western Reserve University and Mass General Brigham (formerly Partners HealthCare). Written informed consent was obtained from all participants.

Acknowledgements: The Cleveland Family Study has been supported in part by National Institutes of Health grants [R01-HL046380, KL2-RR024990, R35-HL135818, and R01-HL113338].

Supplementary Figures

Supplementary Figure 1. *Performance of linear PRSice, LDpred2, and lassosum models.*

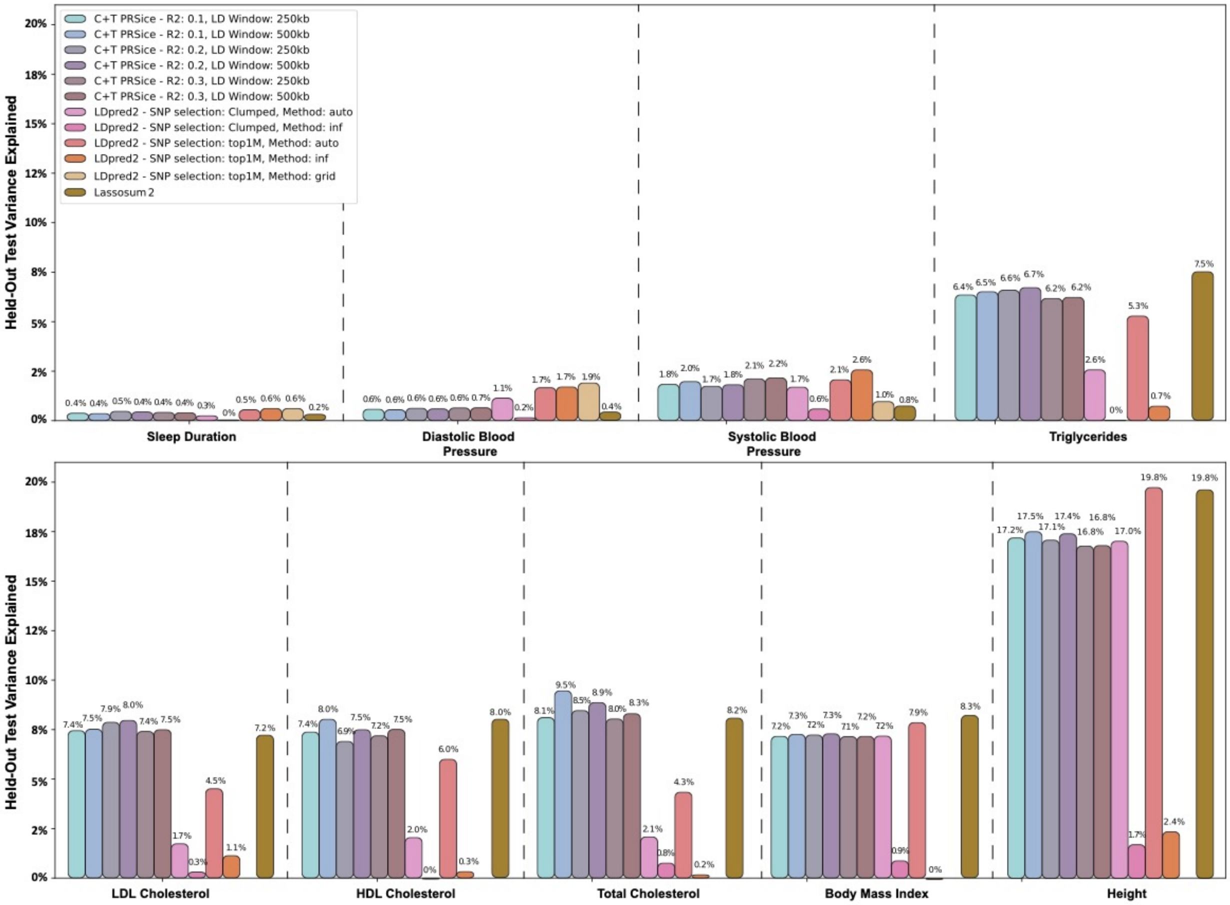

The held-out test set percentage of variance explained for each hyperparameter tested for calculating the C+T PRS using PRSice (optimal p-value for each tuning parameter category), LDpred2, and lassosum.

Supplementary Figure 2. *Heritability of Phenotypes by Race/Ethnicity.*

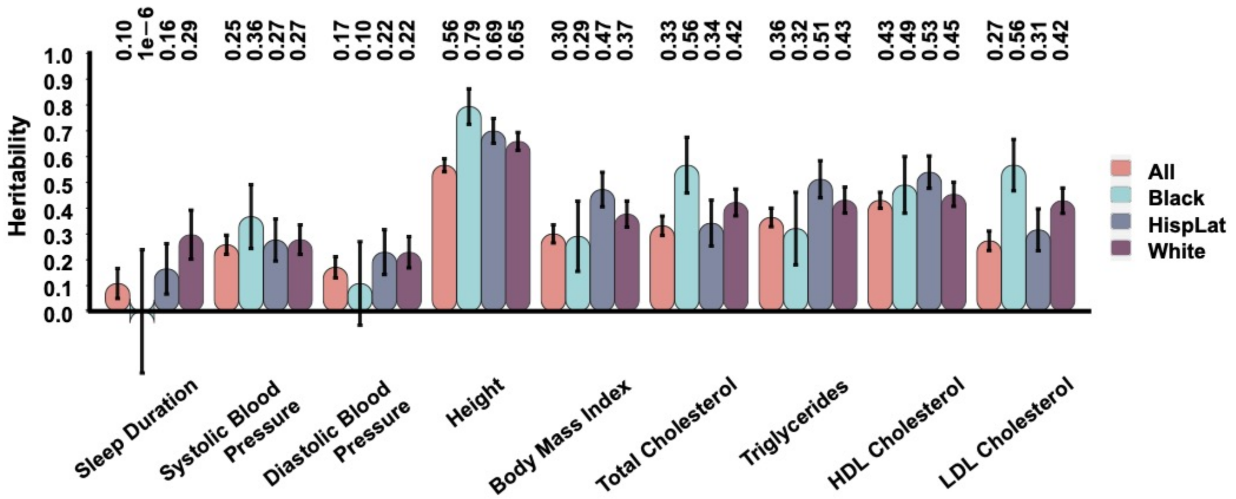

The heritability of each phenotype for each race/ethnic group using the REML approach with error bars of 95% confidence intervals estimated through restricted maximum likelihood estimate

Supplementary Tables

Supplementary Table 1. *Summary statistics of phenotypes in the testing dataset.*

	Black (N=1079)	Hispanic/Latino (N=1447)	White (N=2483)	Overall (N=5009)
**Sex**				
Male	431 (39.9%)	644 (44.5%)	1129 (45.5%)	2204 (44.0%)
Female	648 (60.1%)	803 (55.5%)	1354 (54.5%)	2805 (56.0%)
**Age**				
Mean (SD)	49.4 (17.9)	48.4 (14.3)	54.5 (15.7)	51.6 (16.1)
Median [Min, Max]	52.0 [6.00, 88.0]	49.0 [18.0, 81.0]	55.0 [18.0, 94.0]	53.0 [6.00, 94.0]
**Triglycerides**				
Mean (SD)	114 (109)	132 (88.0)	133 (82.8)	130 (89.3)
Median [Min, Max]	92.0 [21.0, 2040]	112 [24.0, 1510]	113 [18.0, 954]	109 [18.0, 2040]
Missing	488 (45.2%)	257 (17.8%)	556 (22.4%)	1301 (26.0%)
**Total Cholesterol**				
Mean (SD)	202 (38.6)	202 (43.1)	211 (39.2)	207 (40.6)
Median [Min, Max]	199 [112, 360]	198 [83.0, 423]	207 [58.8, 415]	203 [58.8, 423]
Missing	488 (45.2%)	257 (17.8%)	557 (22.4%)	1302 (26.0%)
**Systolic Blood Pressure**				
Mean (SD)	124 (19.6)	121 (17.1)	118 (18.2)	120 (18.4)
Median [Min, Max]	121 [80.5, 225]	118 [85.0, 204]	116 [77.0, 205]	118 [77.0, 225]
Missing	252 (23.4%)	313 (21.6%)	597 (24.0%)	1162 (23.2%)
**Sleep Duration**				
Mean (SD)	6.38 (1.46)	7.77 (1.51)	7.01 (1.18)	7.16 (1.48)
Median [Min, Max]	6.00 [2.00, 14.0]	7.90 [3.00, 13.5]	7.00 [1.00, 12.0]	7.00 [1.00, 14.0]
Missing	281 (26.0%)	79 (5.5%)	1003 (40.4%)	1363 (27.2%)
**Height**				
Mean (SD)	169 (9.83)	163 (9.55)	168 (9.58)	167 (9.91)
Median [Min, Max]	168 [121, 203]	162 [132, 198]	168 [124, 203]	166 [121, 203]
**Diastolic Blood Pressure**				
Mean (SD)	105 (44.5)	91.3 (37.7)	90.3 (39.3)	93.8 (40.5)
Median [Min, Max]	82.0 [0, 256]	76.0 [42.0, 233]	74.0 [28.0, 237]	76.0 [0, 256]
Missing	14 (1.3%)	1 (0.1%)	20 (0.8%)	35 (0.7%)
**HDL Cholesterol**				
Mean (SD)	53.5 (16.1)	49.1 (13.0)	52.0 (15.9)	51.5 (15.2)
Median [Min, Max]	51.0 [20.0, 149]	48.0 [18.0, 119]	50.0 [17.3, 142]	49.1 [17.3, 149]
Missing	24 (2.2%)	4 (0.3%)	35 (1.4%)	63 (1.3%)
**LDL Cholesterol**				
Mean (SD)	122 (35.9)	124 (36.6)	129 (35.3)	126 (36.0)
Median [Min, Max]	119 [22.0, 256]	121 [30.4, 307]	127 [25.0, 334]	123 [22.0, 334]

Supplementary Table 1. *Summary statistics of phenotypes in the testing dataset.*

	**Black (N=1079)**	**Hispanic/Latino (N=1447)**	**White (N=2483)**	**Overall (N=5009)**
Missing	36 (3.3%)	27 (1.9%)	73 (2.9%)	136 (2.7%)
**BMI**				
Mean (SD)	29.3 (6.89)	29.7 (6.19)	26.4 (4.93)	28.0 (5.98)
Median [Min, Max]	28.5 [14.2, 62.0]	28.9 [14.3, 64.0]	25.8 [16.7, 58.4]	27.1 [14.2, 64.0]
Missing	1 (0.1%)	1 (0.1%)	1 (0.0%)	3 (0.1%)

Mean, Median and percent of missing data for the phenotypes (Triglycerides, Total Cholesterol, HDL Cholesterol, LDL Cholesterol, Systolic Blood Pressure, Diastolic Blood Pressure, Sleep Duration, Body Mass Index and Height) and covariates (sex and age) used in this study. All the traits are presented for the whole database as well as broken down by race (Black, White, and Hispanic/Latino). The test set excludes any related individuals above 3rd degree to itself or any of the training datasets.

Supplementary Table 2. *Summary statistics of phenotypes used in a secondary analysis in which the training dataset excluded related individuals.*

	Black (N=5343)	Hispanic/Latino (N=7362)	White (N=12361)	Overall (N=25066)
**Sex**				
Male	2211 (41.4%)	3226 (43.8%)	5664 (45.8%)	11101 (44.3%)
Female	3132 (58.6%)	4136 (56.2%)	6697 (54.2%)	13965 (55.7%)
**Age**				
Mean (SD)	50.4 (17.9)	48.9 (13.9)	54.1 (15.7)	51.8 (15.9)
Median [Min, Max]	53.0 [6.00, 92.0]	50.0 [18.0, 86.0]	55.0 [5.00, 98.0]	53.0 [5.00, 98.0]
**Triglycerides**				
Mean (SD)	107 (74.4)	136 (96.2)	132 (83.2)	129 (86.9)
Median [Min, Max]	91.0 [16.0, 2040]	114 [20.0, 1670]	112 [17.0, 1600]	109 [16.0, 2040]
Missing	2347 (43.9%)	1317 (17.9%)	2781 (22.5%)	6445 (25.7%)
**Total Cholesterol**				
Mean (SD)	202 (40.9)	201 (43.3)	210 (39.1)	206 (41.0)
Median [Min, Max]	198 [81.0, 450]	198 [62.0, 526]	207 [58.8, 594]	203 [58.8, 594]
Missing	2347 (43.9%)	1317 (17.9%)	2782 (22.5%)	6446 (25.7%)
**Systolic Blood Pressure**				
Mean (SD)	125 (20.5)	121 (17.2)	118 (17.9)	120 (18.5)
Median [Min, Max]	121 [73.0, 246]	119 [77.0, 218]	115 [67.0, 227]	118 [67.0, 246]
Missing	1325 (24.8%)	1634 (22.2%)	2882 (23.3%)	5841 (23.3%)
**Sleep Duration**				
Mean (SD)	6.40 (1.45)	7.72 (1.51)	7.00 (1.16)	7.15 (1.45)
Median [Min, Max]	6.00 [1.00, 16.5]	7.79 [2.00, 13.5]	7.00 [1.00, 15.0]	7.00 [1.00, 16.5]
Missing	1477 (27.6%)	398 (5.4%)	4993 (40.4%)	6868 (27.4%)
**Height**				
Mean (SD)	169 (9.54)	163 (9.31)	168 (9.71)	167 (9.89)
Median [Min, Max]	168 [121, 207]	162 [132, 198]	168 [109, 203]	166 [109, 207]
**Diastolic Blood Pressure**				
Mean (SD)	106 (44.8)	91.3 (37.3)	89.8 (38.7)	93.8 (40.2)
Median [Min, Max]	83.0 [0, 267]	76.0 [40.0, 256]	74.0 [23.0, 246]	76.0 [0, 267]
Missing	68 (1.3%)	9 (0.1%)	89 (0.7%)	166 (0.7%)
**HDL Cholesterol**				
Mean (SD)	53.6 (15.4)	49.0 (13.2)	52.0 (16.0)	51.5 (15.2)
Median [Min, Max]	51.0 [15.4, 162]	47.0 [13.0, 141]	49.1 [9.63, 143]	49.0 [9.63, 162]
Missing	146 (2.7%)	10 (0.1%)	167 (1.4%)	323 (1.3%)
**LDL Cholesterol**				
Mean (SD)	123 (37.2)	123 (36.7)	128 (36.1)	126 (36.6)
Median [Min, Max]	120 [11.6, 349]	121 [23.8, 417]	126 [13.8, 505]	123 [11.6, 505]
Missing	186 (3.5%)	145 (2.0%)	328 (2.7%)	659 (2.6%)
**BMI**				

Supplementary Table 2. *Summary statistics of phenotypes used in a secondary analysis in which the training dataset excluded related individuals.*

	Black (N=5343)	Hispanic/Latino (N=7362)	White (N=12361)	Overall (N=25066)
Mean (SD)	29.2 (6.68)	30.0 (6.19)	26.3 (4.79)	28.0 (5.91)
Median [Min, Max]	28.3 [12.7, 75.1]	29.1 [14.3, 70.3]	25.7 [14.4, 58.4]	27.1 [12.7, 75.1]
Missing	4 (0.1%)	8 (0.1%)	6 (0.0%)	18 (0.1%)

Mean, Median and percent of missing data for the phenotypes (Triglycerides, Total Cholesterol, HDL Cholesterol, LDL Cholesterol, Systolic Blood Pressure, Diastolic Blood Pressure, Sleep Duration, Body Mass Index and Height) and covariates (sex and age) used in this study. All the traits are presented for the whole database as well as broken down by race (Black, White, and Hispanic/Latino). This training set excludes any related individuals above 3rd degree.

Supplementary Table 3. *Excluding relatives from the training and validation datasets does not significantly change results.*

Phenotype	**Held-Out Validation PVE for XGBoost Ensemble Model – Excluding Relatives**
Triglycerides	10.3%
Total Cholesterol	14.7%
Systolic Blood Pressure	2.4%
Sleep Duration	0.27%
Height	21.3%

The percentage of variance explained in the validation set when the model is trained and validated on the subset of subjects that are not related (using the kinship coefficient to define related individuals). Sensitivity analyses are limited to phenotypes: Triglycerides, Total Cholesterol, Systolic Blood Pressure, Sleep Duration, and Height.

Supplementary Table 4. *Selected parameters in cross-validation in the main multi-ethnic analysis for PRS and XGBoost*.

Phenotype	XGBoost Alone (and LASSO with XGBoost variants)			XGBoost with PRS			PRS	
	α	SNPs	θ	α	SNPs	θ	Best Performing Model	SNPs
Sleep Duration	0.026	35	316	0.026	36	336	LDpred2 - SNP selection: top1M, Method: auto	1M
Diastolic Blood Pressure	0.367	297	574	0.367	298	555	LDpred2 - SNP selection: top1M, Method: grid	1M
Systolic Blood Pressure	0.336	38	191	0.337	27	309	LDpred2 - SNP selection: top1M, Method: inf	1M
Triglycerides	0.01	186	1028	0.013	109	785	Lassosum	8,035
LDL Cholesterol	0.345	727	1803	0.456	429	1206	C+T PRSice - R2: 0.1, LD Window: 500kb	5825
HDL Cholesterol	0.025	6746	1207	0.21	168	550	C+T PRSice - R2: 0.1, LD Window: 500kb	7694
Total Cholesterol	0.002	1181	1915	0.006	84	720	C+T PRSice - R2: 0.1, LD Window: 500kb	6258
Body Mass Index	0.131	44	497	0.137	51	474	Lassosum	51,113
Height	0.043	4807	4021	0.09	559	706	LDpred2 - SNP selection: top1M, Method: auto	1M

Regularization parameters and number of SNPs selected through cross-validation. α refers to the regularization parameter in the LASSO. θ refers to the number of gradient boosted trees.

Supplementary Table 5. ***Secondary analysis comparing the ensemble model with a standard linear PRS model using the same potential SNP set.***

Phenotype	*C+T PRS with p<1e-4 threshold*	*XGBoost Alone*
Body Mass Index	5.3%	2.9%
Diastolic Blood Pressure	0.5%	1.7%
HDL Cholesterol	8.0%	6.7%
Height	16.2%	8.8%
LDL Cholesterol	7.5%	11.7%
Sleep Duration	0.4%	0.7%
Systolic Blood Pressure	2.0%	2.1%
Total Cholesterol	9.5%	12.7%
Triglycerides	6.5%	8.2%

Linear models using PRS based on SNPs with p-value<10-4 are directly comparable to the XGBoost model as they contain the same set of candidate SNPs.

Supplementary Table 6. ***Comparison of LASSO SNP selection to random SNP selection for the XGBoost Alone and XGBoost with PRS models.***

	Random SNPs XGBoost	***XGBoost Alone (with LASSO SNPs)***	***% Increase***	***Random SNPs XGBoost with PRS***	***XGBoost with PRS (with LASSO SNPs)***	***% Increase***
Total Cholesterol	4.6%	12.7%	176.1%	14.1%	15.5%	9.9%
Triglycerides	4%	8.2%	105.0%	10.5%	10.8%	2.9%
LDL Cholesterol	6.8%	11.7%	72.1%	11%	13.3%	20.9%
HDL Cholesterol	5.5%	6.7%	21.8%	9.5%	10.2%	7.4%

Using random SNP selection instead of the LASSO method for four phenotypes, demonstrates that the LASSO SNP selection improves upon the random baseline by 20%-175% for XGBoost alone. For XGBoost with PRS, the increase is more attenuated at 7%-21%. We performed this experiment by 1) randomly selecting SNPs in the same size as the LASSO selected SNPs for those phenotypes, 2) running the XGBoost model with and without PRS, 3) repeating 100 times, and 4) averaging the result.

Supplementary Table 7. ***Secondary Analysis of Lassosum SNP selection for LDL Cholesterol.***

**Method**	**Test EVR (LDL Cholesterol)**
**XGBoost with LASSO Selected SNPs and PRS**	13.3%
**XGBoost with Lassosum Selected SNPs and PRS**	9.0%

For LDL Cholesterol, we ran the XGBoost algorithm on the SNPs with non-zero weighting from the lassosum algorithm. This is comparable to the XGBoost Alone algorithm with the LASSO selected SNPs.

Supplementary Table 8. *Clumping the SNPs prior to performing the XGBoost ensemble model does not significantly change results.*

Phenotype	**Held-Out Validation PVE for XGBoost Ensemble Model – Clumped SNPs**
Triglycerides	10.6%
Total Cholesterol	14.6%
Systolic Blood Pressure	2.7%
Sleep Duration	0.46%
Height	21.5%

The percentage of variance explained in the validation set when the model is trained on the clumped SNPs rather than all SNPs. Sensitivity analyses are limited to phenotypes: Triglycerides, Total Cholesterol, Systolic Blood Pressure, Sleep Duration, and Height.

Supplementary Table 9. *Results of the PRS model without including the genetic PCs as covariates.*

Phenotype	**Held-Out Validation PVE for PRS Model – Without PCs as Covariates**
Triglycerides	6.2%
Total Cholesterol	8.5%
Systolic Blood Pressure	1.2%
Sleep Duration	0.06%
Height	14.0%

The results are slightly lower than the results of the PRS model that does include the genetic PCs as covariates. Sensitivity analyses are limited to phenotypes: Triglycerides, Total Cholesterol, Systolic Blood Pressure, Sleep Duration, and Height.

Supplementary Table 10. *Results of the LASSO model when performing cross-validation for the optimal regularization term with respect to the LASSO loss function, rather than the joint-training scheme with XGBoost.*

Phenotype	**Held-Out Validation PVE for LASSO Model – Cross-Validation for Optimal Regularization Term**
Triglycerides	6.8%
Total Cholesterol	10.6%
Systolic Blood Pressure	1.6%
Sleep Duration	0.027%
Height	10.5%

Results are slightly higher for the LASSO model but are not directly comparable to the XGBoost models as they include different variants. Sensitivity analyses are limited to phenotypes: Triglycerides, Total Cholesterol, Systolic Blood Pressure, Sleep Duration, and Height.

Supplementary Table 11. *Number of SNPs selected through cross-validation in the race/ethnic-specific XGBoost models.*

Phenotype	***Black***	***Hispanic/Latino***	***White***
Sleep Duration	2541	1771	7009
Diastolic Blood Pressure	129	61	976
Systolic Blood Pressure	29	153	172
Triglycerides	3899	77	715
LDL Cholesterol	248	447	61
HDL Cholesterol	568	284	102
Total Cholesterol	2826	78	55
Body Mass Index	148	38	6230
Height	430	29	207

Number of SNPs selected through cross-validation in the Black, Hispanic/Latino, and White XGBoost models that included the C+T PRS.

Supplementary Table 12: *Phenotypes used in the analyses and their codes*.

**Phenotype**	**Transformation if applied**	**Role**	**Code in the TOPMed phenotype database**
Sex		Adjusting covariate	annotated_sex_1
Age		Adjusting covariate	age_at_height_baseline_1
Race / Ethnicity	Only kept samples where the two race phenotypes agree	Adjusting covariate or stratification	race_us_1
	Only kept samples where the two race phenotypes agree	Adjusting covariate or stratification	hispanic_or_latino_1
Principal Components 1-5		Adjusting covariate	EV1, EV2, EV3, EV4, EV5
Height		Phenotype	height_baseline_1
Body Mass Index		Phenotype	bmi_1
Lipid Lowering medication usage		variable	lipid_lowering_medication_1
Total Cholesterol	Log-transformed, only kept samples where no lipid medication was used. Removed if age at measurement differs from rest	Phenotype	total_cholesterol_1
Low Density Lipoprotein (LDL) Cholesterol	Only kept samples where no lipid medication was used. Removed if age at measurement differs from rest	Phenotype	ldl _1
High Density Lipoprotein (HDL) Cholesterol	Only kept samples where no lipid medication was used. Removed if age at measurement differs from rest	Phenotype	hdl _1
Triglycerides	Log-transformed, only kept samples where no lipid medication was used. Removed if age at measurement differs from rest	Phenotype	triglycerides_1
Blood Pressure medication usage		variable	antihypertensive_meds_1
Systolic Blood Pressure	Only kept samples where no lipid medication was used. Removed if age at measurement differs from rest	Phenotype	bp_systolic_1
Diastolic Blood Pressure	Add +10 to value if blood pressure lowering medications were used . Removed if age at measurement differs from rest	Phenotype	bp_diastolic_1
Sleep Duration	Removed if age at measurement differs from rest	Phenotype	sleep_duration_1

Phenotypes harmonized by TOPMed DCC used in this study either as target phenotypes, covariates, or variables required for exclusion criteria.

Supplementary Table 13. ***Number of variants overlapping between the TOPMed genotype data after QC and filtering and the GWAS data.***

Phenotype	*Variants in GWAS*	*Variants Overlapping between TOPMed Data and GWAS Data*	*Percentage*
Sleep Duration	3,770,613	3,503,570	93%
Diastolic Blood Pressure	8,223,952	5,523,773	67%
Systolic Blood Pressure	6,232,246	6,232,246	100%
Triglycerides	7,790,474	5,398,074	69%
LDL Cholesterol	7,651,584	4,982,169	65%
HDL Cholesterol	7,843,001	5,156,681	66%
Total Cholesterol	7,642,815	5,271,153	69%
Body Mass Index	1,429,222	1,420,646	99%
Height	1,415,952	1,410,726	100%

For about half of the phenotypes, 90% or more of the variants in the GWAS were found in the TOPMed data. For the other half, 60-70% were found.

Supplementary References

1. Lavange, L. M. *et al.* Sample design and cohort selection in the Hispanic Community Health Study/Study of Latinos. *Ann Epidemiol* **20,** 642–649 (2010).
2. Sorlie, P. D. *et al.* Design and implementation of the Hispanic Community Health Study/Study of Latinos. *Ann Epidemiol* **20,** 629–641 (2010).
3. Dawber, T. R., Kannel, W. B. & Lyell, L. P. An approach to longitudinal studies in a community: the Framingham Study. *Ann. N. Y. Acad. Sci.* **107,** 539–556 (1963).
4. Splansky, G. L. *et al.* The Third Generation Cohort of the National Heart, Lung, and Blood Institute's Framingham Heart Study: design, recruitment, and initial examination. *Am. J. Epidemiol.* **165,** 1328–1335 (2007).
5. Kannel, W. B., Feinleib, M., McNamara, P. M., Garrison, R. J. & Castelli, W. P. An investigation of coronary heart disease in families. The Framingham offspring study. *Am. J. Epidemiol.* **110,** 281–290 (1979).
6. The ARIC Investigators. The Atherosclerosis Risk in Communities (ARIC) Study: design and objectives. . *Am. J. Epidemiol.* **129,** 687–702 (1989).
7. Bild, D. E. *et al.* Multi-Ethnic Study of Atherosclerosis: objectives and design. *Am. J. Epidemiol.* **156,** 871–881 (2002).
8. Friedman, G. D. *et al.* CARDIA: study design, recruitment, and some characteristics of the examined subjects. *J. Clin. Epidemiol.* **41,** 1105–1116 (1988).
9. Wyatt, S. B. *et al.* A community-driven model of research participation: the Jackson Heart Study Participant Recruitment and Retention Study. *Ethn Dis* **13,** 438–455 (2003).
10. Taylor, H. A. *et al.* Toward resolution of cardiovascular health disparities in African Americans: design and methods of the Jackson Heart Study. *Ethn Dis* **15,** S6-4 (2005).
