# Supplementary material for: Non-linear machine learning models incorporating SNPs and PRS improve polygenic prediction in diverse human populations
Source: Commun Biol. 2022 Aug 22;5:856. doi: 10.1038/s42003-022-03812-z (PMC9395509; doi:10.1038/s42003-022-03812-z)
Supplement: Supplementary file 3 — Description of Additional Supplementary Files [file 42003_2022_3812_MOESM3_ESM.pdf]

Description of Additional Supplementary Files

File name: Supplementary Data 1
Description: The source data behind the figures in the paper.
