# Supplementary material for: Non-linear machine learning models incorporating SNPs and PRS improve polygenic prediction in diverse human populations
Source: Commun Biol. 2022 Aug 22;5:856. doi: 10.1038/s42003-022-03812-z (PMC9395509; doi:10.1038/s42003-022-03812-z)
Supplement: Supplementary file 5 — Reporting summary [file 42003_2022_3812_MOESM5_ESM.pdf]

nature portfolio

Corresponding author(s): Tamar Sofer

Last updated by author(s): Jul 14, 2022

Reporting Summary

Nature Portfolio wishes to improve the reproducibility of the work that we publish. This form provides structure for consistency and transparency in reporting. For further information on Nature Portfolio policies, see our Editorial Policies and the Editorial Policy Checklist.

Statistics

For all statistical analyses, confirm that the following items are present in the figure legend, table legend, main text, or Methods section.

n/a	Confirmed	
☐	☒	The exact sample size (*n*) for each experimental group/condition, given as a discrete number and unit of measurement
☐	☒	A statement on whether measurements were taken from distinct samples or whether the same sample was measured repeatedly
☒	☐	The statistical test(s) used AND whether they are one- or two-sided<br>*Only common tests should be described solely by name; describe more complex techniques in the Methods section.*
☐	☒	A description of all covariates tested
☐	☒	A description of any assumptions or corrections, such as tests of normality and adjustment for multiple comparisons
☐	☒	A full description of the statistical parameters including central tendency (e.g. means) or other basic estimates (e.g. regression coefficient) AND variation (e.g. standard deviation) or associated estimates of uncertainty (e.g. confidence intervals)
☐	☒	For null hypothesis testing, the test statistic (e.g. *F*, *t*, *r*) with confidence intervals, effect sizes, degrees of freedom and *P* value noted<br>*Give P values as exact values whenever suitable.*
☒	☐	For Bayesian analysis, information on the choice of priors and Markov chain Monte Carlo settings
☒	☐	For hierarchical and complex designs, identification of the appropriate level for tests and full reporting of outcomes
☒	☐	Estimates of effect sizes (e.g. Cohen's *d*, Pearson's *r*), indicating how they were calculated

Our web collection on statistics for biologists contains articles on many of the points above.

Software and code

Policy information about availability of computer code

Data collection	This work uses data generated by TOPMed freeze 8. No specific software was used to collect the data in this analysis. For methods describing data collection by TOPMed please see: https://www.nhlbiwgs.org/topmed-whole-genome-sequencing-methods-freeze-8
Data analysis	Heritability was estimated using the GCTA software (version 1.93.2). We used PRSice 2 software (version 2.3.1), LDPred2 (bigsnpr v 1.9.5), and Lassosum2 (bigsnpr v 1.9.5) to calculate polygenic risk scores. We used lasso package in R as well as Python 3, scikit-learn and the xgboost packages for all the rest.

For manuscripts utilizing custom algorithms or software that are central to the research but not yet described in published literature, software must be made available to editors and reviewers. We strongly encourage code deposition in a community repository (e.g. GitHub). See the Nature Portfolio guidelines for submitting code & software for further information.

Data

Policy information about availability of data

All manuscripts must include a data availability statement. This statement should provide the following information, where applicable:

- Accession codes, unique identifiers, or web links for publicly available datasets
- A description of any restrictions on data availability
- For clinical datasets or third party data, please ensure that the statement adheres to our policy

TOPMed freeze 8 WGS data are available by application to dbGaP according to the study specific accessions: FHS: phs000974.v4.p3, JHS: phs000964.v1.p1, MESA:

phs001211.v3.p2, CARDIA: phs001612.v1.p1, CFS: phs000954.v3.p2, CHS: phs001368.v2.p1, HCHS/SOL: phs001395.v1.p1, ARIC phs001416.v2.p1. Study phenotypes are available from dbGaP from parent studies accession: FHS: phs000007.v32.p13, JHS: phs000286.v6.p2, MESA: phs000209.v13.p3, CARDIA: phs000285.v3.p2, CFS: phs000284.v2.p1, CHS: phs000287.v7.p1, HCHS/SOL: phs000810.v1.p1, ARIC: phs000090.v7.p1. Instructions to generate PRS that were used in this manuscript, i.e. SNP identifiers (chromosome and positions in genome build hg38) and alleles are publicly available in a figshare repository52 https://doi.org/10.6084/m9.figshare.20304135.v1.
Ensemble ML models for each of the phenotypes trained over both multi-ethnic and race/ethnic groups are publicly available in a figshare repository53 https://doi.org/10.6084/m9.figshare.20301423.v1.

Human research participants

Policy information about studies involving human research participants and Sex and Gender in Research.

Reporting on sex and gender	We added a statement: Note that sex was self-reported and verified by chromosomal sex, and therefore biological sex and gender identify in these analyses are the same. Thus, we refer to this variable as “sex”. We also added a statement "Sex-stratified analyses were not performed due to limited sample size."
Population characteristics	These are provided in Table 1.
Recruitment	Design of each study that contributed to TOPMed and recruitment are provided in the Supplementary Materials.
Ethics oversight	We provided a section entitled "Ethics regulation" that lists the IRBs for each study contributing to the TOPMed dataset.

Note that full information on the approval of the study protocol must also be provided in the manuscript.

Field-specific reporting

Please select the one below that is the best fit for your research. If you are not sure, read the appropriate sections before making your selection.

☒ Life sciences ☐ Behavioural & social sciences ☐ Ecological, evolutionary & environmental sciences

For a reference copy of the document with all sections, see nature.com/documents/nr-reporting-summary-flat.pdf

Life sciences study design

All studies must disclose on these points even when the disclosure is negative.

Sample size	We used 34,072 unrelated (3rd degree or less), genotyped participants from the TOPMed dataset including 7,601 non-Hispanic Black participants, 14,142 non-Hispanic<br>White participants, and 7,320 participants of Hispanic/Latino descent.
Data exclusions	Individuals defined by phenotypic values above the 99th quantile and values below the 1st quantile for the phenotype, computed over the multi-ethnic dataset,<br>individuals using phenotype-related medications (lipid lowering medication, blood pressure medications)
Replication	The performance of each of the models was evaluated on a held-out test set.
Randomization	The total (multi-ethnic) dataset and the ethnic-specific datasets were randomized and divided such that 20% of the data was held out as validation sets
Blinding	20% of each dataset was held out as validation set

Reporting for specific materials, systems and methods

We require information from authors about some types of materials, experimental systems and methods used in many studies. Here, indicate whether each material, system or method listed is relevant to your study. If you are not sure if a list item applies to your research, read the appropriate section before selecting a response.

Materials & experimental systems

n/a	Involved in the study
☒	☐ Antibodies
☒	☐ Eukaryotic cell lines
☒	☐ Palaeontology and archaeology
☒	☐ Animals and other organisms
☒	☐ Clinical data
☒	☐ Dual use research of concern

Methods

n/a	Involved in the study
☒	☐ ChIP-seq
☒	☐ Flow cytometry
☒	☐ MRI-based neuroimaging
